# Editorial: The promise of sleep technology

**DOI:** 10.3389/fnins.2026.1849903

**Published:** 2026-05-27

**Authors:** Ambrose A. Chiang, Craig Canapari, Cathy Goldstein, Kelly Baron

**Affiliations:** 1 Sleep Medicine Section, Louis Stokes Cleveland Veterans Administration (VA) Medical Center, Cleveland, OH, United States; 2 Division of Pulmonary, Critical Care and Sleep Medicine, University Hospitals Cleveland Medical Center, Cleveland, OH, United States; 3 Department of Medicine, Case Western Reserve University, Cleveland, OH, United States; 4 Department of Pediatrics, Yale School of Medicine, New Haven, CT, United States; 5 Department of Neurology, University of Michigan, Ann Arbor, MI, United States; 6 Department of Family Medicine and Public Health, University of Utah, Salt Lake City, UT, United States

**Keywords:** consumer sleep tracker, home sleep apnea testing, large language model (LLM), machine learning and AI, obstructive sleep apnea, pediatric sleep, sleep technology, wearable

Drawing inspiration from Dr. William Dement's landmark monograph from the 1990s, “*The promise of sleep*,” we have chosen the title “*The Promise of Sleep Technology*” for this Research Topic (Dement and Vaughan, 1999). This Research Topic seeks to provide a current and comprehensive appraisal of technological advances in sleep medicine, premised on the substantial potential these developments hold for patients with sleep disorders. This editorial synthesizes 11 contributions from the Research Topic and identifies prospective trajectories shaping the future of the field.

## Summary of Research Topic

Recent advances in consumer-grade wearable technology and machine learning (ML) have catalyzed growing interest in automated, longitudinal sleep monitoring within home environments (Holfinger et al., 2025). Emblematic of the expanding work in ambulatory sleep monitoring, Davidson et al. developed and validated a deep-learning model for sleep staging using a chest patch cardiorespiratory wearable among individuals with comorbid insomnia and depressive symptoms undergoing sleep restriction therapy. Additionally, Lee et al. investigated the relationship between wearable-derived heart rate variability (HRV) and sleep quality, focusing on wake-after-sleep-onset (WASO) to measure sleep fragmentation. The low-frequency to high-frequency (LF/HF) ratio emerged as the most robust correlate with WASO, supporting its potential as a digital biomarker for sleep quality prediction. In younger aged populations, Hilmisson et al. assessed trends in cyclic alternating pattern-linked cardiopulmonary coupling calculated Sleep Quality Index from childhood through adolescence. The authors found that children seem to reach their full potential in sleep stability and quality around school age, but they start to gradually decline in early adolescence.

In the domain of obstructive sleep apnea (OSA), Nokes et al. sought to elucidate the mechanisms underlying the heterogeneous response to weight loss in OSA, finding that upper airway length was strongly associated with apnea hypopnea index (AHI), hypoxic burden, and ventilatory burden, whereas tongue fat percentage showed no significant association with AHI. Goldstein et al. reported performance of the SANSA device (Huxley Medical, Inc., Atlanta, GA), a novel chest sensor integrating electrocardiogram, photoplethysmography, oximetry, acoustic sensor, and accelerometry, demonstrating robust diagnostic accuracy for OSA detection. Hu et al. assessed the relationship between neurocognitive function and elevated low-frequency narrow band, a high loop gain biomarker that may be a clinically useful predictor of cognitive improvement following CPAP initiation. Ingolfsdottir et al. reported that wearable home sleep testing was able to detect undiagnosed OSA in a sample of obese children aged 4–9, highlighting that contemporary wearable platforms hold promise in diagnosing more children for OSA.

Sleep monitoring does not exist in a silo and has important ramifications for other disorders, including mental health conditions. Defillo, Grassi, Daccò, Martin, Guadagni et al. investigated odds ratio product (ORP) across sleep stages and its association with depressive symptoms in a large clinical cohort, demonstrating that ORP holds promise as a non-invasive biomarker for depressive symptoms. Defillo, Grassi, Daccò, Martin, Caldirola et al. also evaluated the screening accuracy of a novel software, MEB-001, for detecting current major depressive episode (cMDE) in individuals identified as with and without suspected comorbid insomnia and sleep apnea (COMISA), finding that MEB-001 achieved consistent cMDE screening performance comparable to the Patient Health Questionnaire, 9 items (PHQ-9).

Finally, despite the widespread adoption of consumer sleep-tracking applications, the sociodemographic and psychological correlates of their use remain incompletely characterized. Pallesen et al. studied 940 young adults residing in the UK, identifying positive associations with age, insomnia symptoms, and the personality trait of conscientiousness. Lastly, Kakazu et al. conducted a systematic evaluation of industry sponsorship bias in randomized controlled trials of digital Cognitive Behavioral Therapy for Insomnia, revealing a significant association between elevated risk of sponsorship bias and open-access publication status, as well as an inverse relationship between sponsorship bias risk and overall methodological rigor.

## Future trends

Consumer-grade wearable sleep tracking (CWST) has opportunities for exponential growth in the coming years. An ample body of evidence has characterized the performance of CWST devices, and international organizations, including the Sleep Research Society and the World Sleep Society, have issued frameworks to guide their application in research and clinical contexts (Chee et al., 2025; de Zambotti et al., 2024). Nevertheless, CWST has largely been employed to approximate legacy polysomnography (PSG)-derived sleep metrics and are primarily considered alternatives to actigraphy. Such paradigm fails to capitalize on their distinctive capabilities of these devices, including cardiac autonomic monitoring alongside motion sensing, near-real-time data transfer and processing, and simultaneous capture of multiple health data streams (objective and self-reported) that could allow for more cutting-edge applications. For example, sleep estimates derived from movement and cardiac autonomic signals are not equivalent to EEG-defined sleep, yet may yield distinct and complementary insight into sleep physiology and changes related to prevalent and incident health conditions.

Similarly, while some consumer wearables can now stratify risk for OSA, their signals may also enable modeling of cardiac autonomic responses to treatment, distinguishing responders from non-responders and potentially identifying whether therapy is likely to reduce future OSA-associated sequelae, including cardiovascular disease. Moreover, by integrating sleep parameters with self-reported symptoms and contextual data, CWST paired with app-delivered behavioral treatment could support just-in-time adaptive interventions, further refined through reinforcement learning approaches, advancing personalized care for insomnia. Implementation studies are desperately needed to realize the full potential of CWST while systematically identifying potential undesired consequences.

In the domain of OSA assessment, emerging sleep technologies are poised to transform clinical sleep medicine practice across four distinct scenarios in the years to come (Table 1) (Chiang et al., 2024). With respect to OSA risk stratification, two smartwatch-integrated sleep apnea detection algorithms received FDA clearance in 2024, a trajectory likely to continue (Schutte-Rodin et al., 2025). Complementary digital modalities, including large language model (LLM) chatbots, hold promise for disease triaging, yet warrant further validation (Kopka et al., 2025). In primary care environments, ML algorithms can interrogate clinical, demographic, and anthropometric data within electronic health records to identify patients at elevated OSA risk. Among those flagged, diagnosis can be expedited through medical-grade OSA-detecting wearables in medically uncomplicated OSA patients (Lee et al., 2024). For individuals with significant comorbidities or diagnostic uncertainty, attended in-lab PSG remains the gold standard, with level II flow-based home sleep apnea testing (HSAT) serving as a viable alternative in select sleep specialty contexts (Kapur et al., 2017). For patients undergoing therapeutic interventions, longitudinal OSA monitoring and therapeutic response evaluation can potentially be facilitated through OSA-detecting wearables or contactless nearable platforms that support multi-night assessments to account for night-to-night variability (Lechat et al., 2022; Tsai et al., 2026).

**Table 1:** Potential applications of various sleep technologies in distinct clinical settings.

Technology Category	Community: at-home OSA risk stratification	Primary care: screening and diagnosis of uncomplicated OSA	Sleep specialty: advanced diagnosis and management of sleep disorders	Multidisciplinary contexts: longitudinal therapeutic monitoring
Technology accessible to patients				
LLM chatbot or symptom checkers	**++**			
SmartWatch OSA risk assessment	**+++**			
Smartphone (acoustic ± sonar)	**+**			
Nearables (radar or BCG)	**+**			**++**
Technology accessible to clinicians				
ML algorithms using clinical features		**+++**	**+**	
Medical-grade OSA-detecting wearables		**+++**	**+++**	**+++**
Flow-based HSAT		**++**	**+++**	**+**
In-lab PSG		**++**	**+++**	**+**
Overnight pulse oximetry		**+**		**++**

Potential usefulness: +, least useful; +++, most useful.

BCG, ballistocardiogram; HSAT, home sleep apnea testing; LLM, large language model; ML, machine learning; OSA, obstructive sleep apnea; PSG, polysomnography.

Pediatric sleep medicine also represents a particularly dynamic area of clinical investigation. HSAT is not currently endorsed by the American Academy of Sleep Medicine for the diagnosis of pediatric OSA (Patil et al., 2023). Nevertheless, a recent systematic review indicates that level III HSAT demonstrates adequate sensitivity for detecting moderate-to-severe OSA in children, though its ability to reliably detect or exclude mild OSA remains limited (Landry et al., 2024). Another emerging frontier concerns the role of digital pediatric sleep intervention to aid parents in managing their child's sleep. A recent scoping review examining digital tools applied to early childhood sleep health suggests that such technologies hold potential for improving sleep outcomes in children aged 3–8 (Chung et al., 2026). Nonetheless, further rigorous investigation is warranted in this domain.

## Conclusion

This special *Frontiers* issue underscores the transformative potential of emerging sleep technologies, which present unprecedented opportunities for the diagnosis and management of sleep disorders. Capitalizing this momentum, however, necessitates the continued generation of evidence-based research, alongside sustained interdisciplinary collaboration. Ultimately, the promise of sleep technology will be realized through a unified commitment, ensuring that future advances equitably improve sleep health.

## References

[B1] Chee M. W. Baumert M. Scott H. Cellini N. Goldstein C. Baron K. . (2025). World Sleep Society recommendations for the use of wearable consumer health trackers that monitor sleep. Sleep Med. 131:106506. doi: 10.1016/j.sleep.2025.106506 40300398

[B2] Chiang A. A. Jerkins E. Holfinger S. Schutte-Rodin S. Chandrakantan A. Mong L. . (2024). OSA diagnosis goes wearable: are the latest devices ready to shine? J. Clin. Sleep Med. 20, 1823–1838. doi: 10.5664/jcsm.11290 39132687 PMC11530974

[B3] ChungA. DeatonL. MillerJ. NechybaA. LiuJ. MetayerM. . (2026). Handling Editor: Monica AndersenA global perspective of parent engagement with digital sleep health interventions for young children: a scoping review. Sleep Med. Rev. 85:102226. doi: 10.1016/j.smrv.2025.10222641478062

[B4] de Zambotti M. Goldstein C. Cook J. Menghini L. Altini M. Cheng P. . (2024). State of the science and recommendations for using wearable technology in sleep and circadian research. Sleep 47:zsad325. doi: 10.1093/sleep/zsad325 38149978

[B5] Dement W. C. Vaughan C. (1999). The Promise of Sleep: A Pioneer in Sleep Medicine Explores the Vital Connection between Health, Happiness, and a Good Night's Sleep. New York, NY: Delacorte Press.

[B6] Holfinger S. Schutte-Rodin S. Ratnasoma D. Chiang A. A. Baron K. Deak M. . (2025). Evolving trends in novel sleep tracking and sleep testing technology publications between 2020 and 2022. J. Clin. Sleep Med. 21, 891–905. doi: 10.5664/jcsm.11562 39789983 PMC12048328

[B7] Kapur V. K. Auckley D. H. Chowdhuri S. Kuhlmann D. C. Mehra R. Ramar K. . (2017). Clinical practice guideline for diagnostic testing for adult obstructive sleep apnea: an American academy of sleep medicine clinical practice guideline. J. Clin. Sleep Med. 13, 479–504. doi: 10.5664/jcsm.6506 28162150 PMC5337595

[B8] Kopka M. von Kalckreuth N. Feufel M. A. (2025). Accuracy of online symptom assessment applications, large language models, and laypeople for self-triage decisions. NPJ Dig. Med. 8:178. doi: 10.1038/s41746-025-01566-6 40133390 PMC11937345

[B9] Landry V. Semsar-Kazerooni K. Chen T. Gurberg J. Nguyen L. H. P. Constantin E. (2024). Diagnostic accuracy of portable sleep monitors in pediatric sleep apnea: a systematic review. Sleep Med. Rev. 78:101991. doi: 10.1016/j.smrv.2024.101991 39173472

[B10] Lechat B. Naik G. Reynolds A. Aishah A. Scott H. Loffler K. A. . (2022). Multinight prevalence, variability, and diagnostic misclassification of obstructive sleep apnea. Am. J. Respir. Crit. Care Med. 205, 563–569. doi: 10.1164/rccm.202107-1761OC 34904935 PMC8906484

[B11] Lee P. L. Gu W. Huang W. C. Chiang A. A. (2024). “From screening at clinic to diagnosis at home: how AI/ML/DL algorithms are transforming sleep apnea detection,” in Handbook of AI and Data Sciences for Sleep Disorders, eds. R. B. Berry, P.M. Pardalos, and X. Xian (Cham: Springer Nature), 109–160. doi: 10.1007/978-3-031-68263-6_4

[B12] Patil S. Ayas N. Kimoff R. J. Owen R. Paruthi S. Pien G. . (2023). “Obstructive sleep apnea, pediatric.” in International Classification of Sleep Disorders, 3rd Edn, text revision, eds. M. Sateia, R. Chervin, K. Maski, and S. Patil (Darien, IL: American Academy of Sleep Medicine), 79–86.

[B13] Schutte-Rodin S. Holfinger S. Chiang A. A. Bandyopadhyay A. (2025). Consumer sleep technologies that self-screen or self-assess risk for OSA: is subclinical OSA (SCOSA) coming to our clinics? J. Clin. Sleep Med. 21, 1325–1326. doi: 10.5664/jcsm.11738 40219985 PMC12225266

[B14] Tsai C. W. Palomo J. M. Link B. Chen P.-L. Cheung C. Leung L. . (2026). Using a wearable's multi-night capability to mitigate night-to-night variability in a dental clinic cohort. J. Dent. Sleep Med. 13:7416. doi: 10.15331/jdsm.7416

